# Circulating TNF Receptors Are Significant Prognostic Biomarkers for Idiopathic Membranous Nephropathy

**DOI:** 10.1371/journal.pone.0104354

**Published:** 2014-08-06

**Authors:** Su Mi Lee, SeungHee Yang, Ran-hui Cha, Myounghee Kim, Jung Nam An, Jin Ho Paik, Dong Ki Kim, Shin-Wook Kang, Chun Soo Lim, Yon Su Kim, Jung Pyo Lee

**Affiliations:** 1 Department of Internal Medicine, Seoul National University Hospital, Seoul, Korea; 2 Department of Internal Medicine, Dong-A University, Busan, Korea; 3 Kidney Research Institute, Seoul National University, Seoul, Korea; 4 Department of Internal Medicine, National Medical Center, Seoul, Korea; 5 Department of Dental Hygiene, College of Health Science, Eulji University, Seongnam, Korea; 6 Department of Internal Medicine, Seoul National University Boramae Medical Center, Seoul, Korea; 7 Department of Pathology, Seoul National University Bundang Hospital, Seongnam, Korea; 8 Severance Biomedical Science Institute, Brain Korea 21 and Department of Internal Medicine, Yonsei University, Seoul, Korea; Fondazione IRCCS Ospedale Maggiore Policlinico & Fondazione D’Amico per la Ricerca sulle Malattie Renali, Italy

## Abstract

Idiopathic membranous nephropathy (iMN) is a common cause of nephrotic syndrome in adults. A biomarker to accurately indicate the severity of iMN and predict long-term prognosis is insufficient. Here, we evaluated the clinical significance of circulating tumor necrosis factor receptors (cTNFRs) as prognostic biomarkers of iMN with nephrotic syndrome. A total of 113 patients with biopsy-proven iMN and 43 healthy volunteers were enrolled in this study. Ninety patients with iMN had nephrotic range proteinuria. Levels of cTNFRs were measured by using serum samples collected at the time of initial diagnosis. Levels of cTNFRs were higher in the patients with nephrotic syndrome than in those with subnephrotic range proteinuria or in the healthy volunteers (*P* for trend <0.001). Estimated glomerular filtration rate and proteinuria tended to worsen as the cTNFRs levels increased. Having a cTNFR1 level within the highest tertile was a significant risk factor for renal progression after adjustment, in comparison with the other tertiles (hazard ratio [HR], 3.39; 95% confidence interval [95% CI], 1.48–7.78; *P* = 0.004). The cTNFR2 level within the highest tertile also significantly increased the risk of renal progression (HR, 3.29; 95% CI, 1.43–7.54; *P* = 0.005). Renal tubular TNFRs expression was associated with cTNFRs level. However, the cTNFRs levels were not associated with autoantibody against phospholipase A_2_ receptor reactivity/levels or treatment response. This study demonstrated that cTNFRs levels at the time of initial diagnosis could predict renal progression in patients with iMN.

## Introduction

Idiopathic membranous nephropathy (iMN) is a main cause of nephrotic syndrome in adults [1], [2]. Various courses of iMN make it difficult to determine whether treatment should be introduced or what kind of treatment approach is appropriate [3], [4], [5]. Heavy proteinuria, renal dysfunction, and severe histopathological lesions are known as risk factors for renal progression [3], [6], [7], [8]. Recently, autoantibody against phospholipase A_2_ receptor (anti-PLA_2_R) has been accepted as a primary mechanism of iMN accounting for the presence of immune complexes in the glomerular capillary wall [9]. However, this marker could not also completely explain diverse clinical courses of iMN. Additional biomarkers for the prediction of renal progression or treatment response are needed.

Exposure to inflammation is thought to be closely correlated to the development and progression of renal injury [10], [11], [12], [13]. In particular, tumor necrosis factor α (TNFα) has an important role in kidney disease. It is a pleiotropic cytokine with proinflammatory and immunoregulatory properties [14], [15], and its actions are relayed by two distinct TNF receptors (TNFRs), TNFR1 and TNFR2. In IgA nephropathy, TNFRs were found to be up-regulated by TNFα and to induce tubulointerstitial damage, ultimately leading to renal impairment [16]. In addition, circulating TNFRs (cTNFRs) levels were suggested to be significantly associated with progressive nephropathy in type 1 and 2 diabetes [17], [18]. However, the role of cTNFRs in iMN has not been reported. We hypothesized that cTNFRs levels are helpful in the assessment of the initial activity of iMN and a subsequent treatment response.

In this study, we aimed to identify the association between cTNFRs levels at the time of initial diagnosis and clinical manifestations. We also evaluated the role of cTNFRs on the progression of renal function and treatment response.

## Results

### Characteristics of Study Participants

The baseline characteristics of the study participants are shown in Table 1. The participants were stratified into three groups: healthy volunteers (*n* = 43), patients with a subnephrotic range proteinuria (*n* = 23), and patients with nephrotic syndrome (*n* = 90). Mean age, serum total cholesterol level, and proteinuria were higher and mean serum albumin level was lower in the patients with nephrotic syndrome than in the other groups. However, sex, serum creatinine (sCr) level, and estimated glomerular filtration rate (eGFR) did not significantly differ among the three groups. In addition, no significant differences in pathological stage and presence of hypertension were observed between the patients with subnephrotic proteinuria and those with nephrotic syndrome.

**Table 1:** Baseline characteristics of the participants.

	HealthyVolunteer(*n* = 43)	Patients withSubnephroticproteinuria (*n* = 23)	Nephroticsyndrome (*n* = 90)	*P* value
Age (years)	36.9±9.9	49.6±14.1^b^	55.9±13.5^b^	<0.001^a^
Male (n/%)	21 (48.8%)	13 (56.5%)	49 (54.4%)	0.583
Hypertension (n/%)		6 (26.1%)	37 (41.1%)	0.185
Serum creatinine (mg/dL)	0.88±0.16	0.86±0.17	0.94±0.32	0.251
eGFR (mL/min/1.73 m^2^)	92.7±13.5	91.8±19.8	85.0±23.9	0.095
Serum albumin (g/dL)	4.5±0.2	3.8±0.5^b^	2.5±0.5^b^	<0.001^a^
Serum total cholesterol(mg/dL)	173.0±29.2	202.6±50.0	285.5±93.5^b^	<0.001^a^
UPCR (g/g Cr)	0.01±0.03	1.41±0.82	7.77±3.77^b^	<0.001^a^
Pathologic stage (n/%)				0.211
1		5 (21.7%)	23 (25.6%)	
2		12 (52.2%)	44 (48.9%)	
3		4 (17.4%)	22 (24.4%)	
4		2 (8.7%)	1 (1.1%)	

Continuous data are expressed as the mean ± SD and categorical data are expressed as numbers (percentage).

a *P* value for trend <0.001 (analysis of variance with Scheffe’s multiple comparison test).

b *P* value <0.05 compared with healthy volunteer. Analysis of variance with Scheffe’s multiple comparison test was used.

Abbreviations: eGFR, estimated glomerular filtration rate; UPCR, urinary protein to creatinine ratio; SD, standard deviation.

### Association of Clinical Parameters with Circulating TNFRs Levels

**Figure 1:**
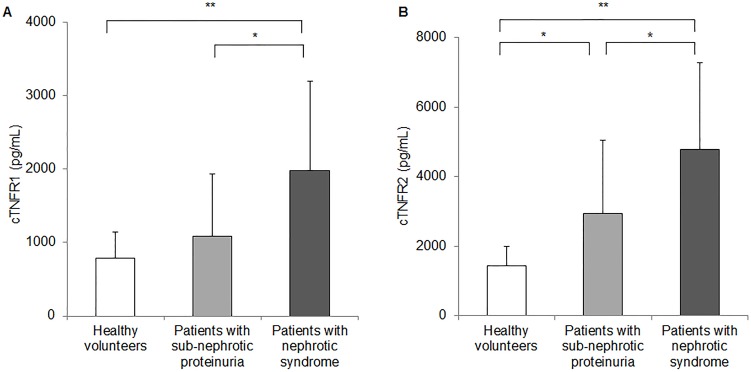
Circulating TNFRs levels in the patients with iMN compared with those in the healthy volunteers. **P<0.001, *P<0.05.

The mean cTNFRs levels were most dominant in the patients with nephrotic syndrome. The mean cTNFR1 level was higher in the patients with nephrotic syndrome than in those with subnephrotic proteinuria or in the healthy volunteers (cTNFR1: nephrotic syndrome group vs. subnephrotic proteinuria group vs. healthy volunteers, 1976.8±1217.6 pg/mL vs. 1086.2±854.1 pg/mL vs. 789.3±351.1 pg/mL, *P* for trend <0.001; Figure 1A). Moreover, the mean cTNFR2 level in the patients with nephrotic syndrome was higher than in those with subnephrotic proteinuria or in the healthy volunteers (cTNFR2: nephrotic syndrome group vs. subnephrotic proteinuria group vs. healthy volunteers, 4777.8±2499.3 pg/mL vs. 2949.7±2104.5 pg/mL vs. 1438.1±565.8 pg/mL, *P* for trend <0.001; Figure 1B).

The cTNFRs levels and clinical parameters were significantly related (Table 2). The patients with nephrotic syndrome were categorized based on cTNFRs levels tertiles. The eGFR worsened proportionally with the increase in the cTNFRs. The patients with cTNFR1 level within the highest tertile had older age, higher sCr level, proteinuria, hypertension, and advanced pathologic stage but lower eGFR and albumin level than those with cTNFR1 level within the other tertiles. A similar result was found in the relationship between cTNFR2 and clinical parameters. Estimated GFR showed significant negative correlation with log-transformed cTNFRs (Ln cTNFRs) levels (Pearson’s correlation coefficient (*r*) = −0.571, *P*<0.001 for Ln cTNFR1 and *r* = −0.466, *P*<0.001 for Ln cTNFR2; Figure S1A). Proteinuria level was also positively correlated with Ln cTNFRs, but the degree of correlation was lesser than eGFR (*r* = 0.240, *P* = 0.024 for Ln cTNFR1; *r* = 0.196, *P* = 0.066 for Ln cTNFR2; Figure S1B).

**Table 2:** Correlation between circulating TNFRs levels and clinical parameters.

	cTNFR1 T1	cTNFR1 T2	cTNFR1 T3	*P* value
Age (years)	53.8±12.1	50.7±12.4	63.0±13.6^b^	0.001^a^
Male (n/%)	12 (25.0%)	20 (41.7%)	16 (33.3%)	0.369
Hypertension (n/%)	8 (22.2%)	10 (27.8%)	18 (50.0%)	0.011^a^
Serum creatinine (mg/dL)	0.77±0.16	0.88±0.17	1.16±0.42^b^	<0.001^a^
eGFR (mL/min/1.73 m^2^)	98.1±19.1	91.1±16.1	67.2±23.8^b^	<0.001^a^
Serum albumin (g/dL)	2.7±0.5	2.5±0.5	2.3±0.5^b^	0.006^a^
Serum total cholesterol (mg/dL)	275.1±75.3	311.8±93.1	272.2±106.2	0.202
UPCR (g/g Cr)	7.52±3.98	6.61±3.00	9.27±3.90	0.020^a^
Pathologic stage (n/%)				0.004^a^
1–2	25 (29.8%)	25 (29.8%)	16 (19.0%)	
3–4	4 (13.8%)	5 (17.2%)	14 (48.3%)	
	**cTNFR2 T1**	**cTNFR2 T2**	**cTNFR2 T3**	***P*** ** value**
Age (years)	54.8±12.4	50.5±13.1	62.2±13.1	0.003^a^
Male (n/%)	13 (27.1%)	19 (39.6%)	16 (33.3%)	0.523
Hypertension (n/%)	7 (19.4%)	11 (30.6%)	18 (50.0%)	0.005^a^
Serum creatinine (mg/dL)	0.80±1.96	0.90±0.21	1.11±0.43^b^	<0.001^a^
eGFR (mL/min/1.73 m^2^)	96.3±20.4	88.7±17.9	71.4±25.7^b^	<0.001^a^
Serum albumin (g/dL)	2.76±0.53	2.33±0.45^b^	2.39±0.47^b^	0.002^a^
Serum total cholesterol (mg/dL)	287.5±80.9	303.0±107.8	270.0±90.9	0.412
UPCR (g/g Cr)	7.26±3.97	6.68±3.17	9.46±3.69	0.009^a^
Pathologic stage (n/%)				0.022^a^
1–2	24 (28.6%)	25 (29.8%)	17 (20.2%)	
3–4	5 (17.2%)	5 (17.2%)	13 (44.8%)	

Continuous data are expressed as the mean ± SD and categorical data are expressed as numbers (percentage).

a *P* value for trend <0.05 (analysis of variance with Scheffe’s multiple comparison test).

b *P* value <0.05 compared with TNFRs T1 subgroup. Analysis of variance with Scheffe’s multiple comparison test was used.

Abbreviations: eGFR, estimated glomerular filtration rate; UPCR, urinary protein to creatinine ratio; cTNFR1, circulating tumor necrosis factor receptor 1; cTNFR2, circulating tumor necrosis factor receptor 2; SD, standard deviation.

### Association of Histological Features with Circulating TNFRs Levels

**Figure 2:**
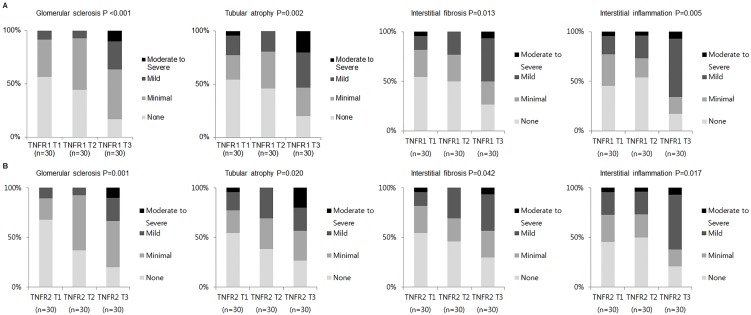
Proportion of the patients based on severity of pathological findings according to circulating TNFRs levels. (A) Correlation between the pathological findings and the circulating TNFR1 tertiles. (B) Correlation between the pathological findings and circulating TNFR2 tertiles.

In the patients with nephrotic syndrome, a significant association was observed between the cTNFR1 level and histological features, including glomerular sclerosis, tubular atrophy, interstitial fibrosis, and interstitial inflammation (Figure 2A). When histological features were scored as 0 to 3, glomerular sclerosis, tubular atrophy, interstitial fibrosis, and interstitial inflammation tended to be more severe with every cTNFR1 tertile increment (*P*<0.001, *P* = 0.002, *P* = 0.013, and *P* = 0.005, respectively). Furthermore, these findings were observed in the association between cTNFR2 level and worsening of glomerular sclerosis, tubular atrophy, interstitial fibrosis, and interstitial inflammation (*P* = 0.001, *P* = 0.020, *P* = 0.042, and *P* = 0.017, respectively; Figure 2B).

### Association of Treatment Response with Circulating TNFRs Levels

We evaluated the potential of cTNFRs levels as predictive biomarkers of treatment response in patients with nephrotic syndrome. The mean follow-up time was 41.6±35.3 months. While 23 patients (25.6%) received only conservative treatment, 65 patients (72.2%) received at least four specific treatments with an immunosuppressant such as glucocorticoid combined with cyclophosphamide (28.9%), glucocorticoid combined with calcineurin inhibitors (20.0%), glucocorticoid alone (20.0%), and glucocorticoid combined with mycophenolate mofetil (2.2%).

Of all the patients, 85.6% achieved remission, either a complete remission (CR) or a partial remission (PR), at a mean time of 11.7±10.2 months. No significant differences in clinicopathological findings were found between the remission and non-responder groups (Table 3). The Ln cTNFRs levels were not associated with responsiveness to treatment (non-responder group vs. remission group, 7.58±0.77 pg/mL vs. 7.40±0.52 pg/mL for Ln cTNFR1, *P* = 0.288; 8.43±0.67 pg/mL vs. 8.33±0.48 pg/mL for Ln cTNFR2, *P* = 0.509).

**Table 3:** Comparison of the clinical characteristics according to presence of remission.

	Remission	Non-responder	*P* value
Number of patients	77	13	
Age (years)	55.4±12.9	58.8±17.3	0.513
Male (n/%)	42 (54.5%)	7 (53.8%)	0.963
Hypertension (n/%)	36 (40.0%)	3 (21.4%)	0.182
Serum creatinine (mg/dL)	0.92±0.28	1.05±0.51	0.400
eGFR (mL/min/1.73 m^2^)	85.6±22.7	81.3±31.0	0.548
Serum albumin (g/dL)	2.5±0.5	2.4±0.3	0.348
Serum total cholesterol (mg/dL)	289.0±94.4	263.8±88.4	0.390
UPCR (g/g Cr)	7.58±3.58	8.84±4.78	0.270
Glomerular sclerosis	0.84±0.80	0.92±0.90	0.765
Tubular atrophy	1.04±1.01	1.17±1.12	0.705
Interstitial fibrosis	0.94±0.94	0.92±0.90	0.936
Interstitial inflammation	1.06±0.99	1.17±0.84	0.728
Ln cTNFR1 (pg/mL)	7.40±0.52	7.58±0.77	0.288
Ln cTNFR2 (pg/mL)	8.33±0.48	8.43±0.67	0.509

Continuous data are expressed as the mean ± SD and categorical data are expressed as numbers (percentage).

Abbreviations: eGFR, estimated glomerular filtration rate; UPCR, urinary protein to creatinine ratio; Ln cTNFR1, log-transformed circulating tumor necrosis factor receptor 1; Ln cTNFR2, log-transformed circulating tumor necrosis factor receptor 2; SD, standard deviation.

### Prediction of Renal Progression by Circulating TNFRs Levels: Multivariable Models

**Figure 3:**
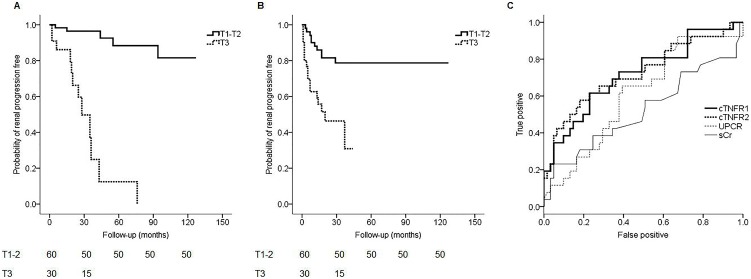
Kaplan-Meier survival curves of renal progression. (A) The patients with cTNFR1 level within the highest tertile had a significantly faster renal progression compared with those with cTNFR1 level within the other tertiles (*P*<0.001, log-rank test). (B) The patients with cTNFR2 level within the highest tertile also had a significantly faster renal progression compared with those with cTNFR2 level within the other tertiles (*P*<0.001, log-rank test). (C) ROC curves for circulating TNFR1 (cTNFR1), circulating TNFR2 (cTNFR2), UPCR, and serum creatinine (sCr) determining renal progression. The AUCs for cTNFR1, cTNFR2, sCr, and UPCR were 0.719 (95% confidence interval [95% CI]: 0.597–0.841), 0.724 (95% CI: 0.599–0.849), and 0.520 (95% CI: 0.375–0.666), 0.607 (95% CI: 0.479–0.735), respectively.

The impact of cTNFRs expression on renal progression was evaluated by using a Kaplan-Meier analysis (Figure 3A and 3B). The risk of renal progression during the follow-up rapidly increased in the patients with cTNFR1 level within the highest tertile compared with those with cTNFR1 level within the other tertiles (*P*<0.001, log-rank test). Similarly, the patients with cTNFR2 level within the highest tertile had a significantly faster renal progression compared with those with cTNFR2 level within the other tertiles (*P*<0.001, log-rank test).

The independent effect of cTNFRs on renal progression was examined by using multivariate Cox proportional hazard models (Table 4). The highest cTNFR1 tertile remained as an independent variable associated with renal progression after adjustment for all of the confounding variables, including age, sex, presence of hypertension, eGFR, proteinuria, pathologic stage, kind of treatment, and presence of remission. The patients with TNFR1 level within the highest tertile were more than 3 times more likely to progress to renal dysfunction than those with cTNFR1 level within the other tertiles (hazard ratio [HR]: 3.39, 95% CI: 1.48–7.78, *P* = 0.004). Furthermore, a similar result was identified for cTNFR2 level (HR: 3.29, 95% CI: 1.43–7.54, *P* = 0.005).

**Table 4:** Multivariate Cox proportional model for renal function progression.

	Univariate		Model 1		Model 2		Model 3	
	HR (95% CI)	*P* value	HR (95% CI)	*P* value	HR (95% CI)	*P* value	HR^a^ (95% CI)	*P* value
cTNFR1 (pg/mL)								
T1–2 (reference)	1.00		1.00		1.00		1.00	
T3	4.49 (2.02–10.02)	<0.001	4.49 (2.02–10.02)	<0.001	4.50 (2.02–10.02)	<0.001	3.39 (1.48–7.78)^b^	0.004
cTNFR2 (pg/mL)								
T1–2 (reference)	1.00		1.00		1.00		1.00	
T3	4.09 (1.83–9.11)	0.001	4.09 (1.83–9.11)	0.001	4.09 (1.83–9.11)	0.001	3.29 (1.43–7.54)^b^	0.005
Age (years)	1.03 (1.00–1.06)	0.460	1.01 (0.98–1.04)	0.443	1.02 (1.00–1.05)	0.275	1.00 (0.96–1.04)	0.932
Male	1.53 (0.71–3.31)	0.282	1.54 (0.71–3.34)	0.272	1.44 (0.65–3.16)	0.366	2.19 (0.93–5.16)	0.073
Hypertension	1.75 (0.81–3.79)	0.156			1.17 (0.48–2.83)	0.731	1.39 (0.56–3.42)	0.477
eGFR (mL/min/1.73 m^2^)	0.99 (0.98–1.01)	0.380			1.01 (0.99–1.03)	0.372	1.00 (0.98–1.03)	0.680
UPCR (g/g Cr)	1.10 (1.00–1.20)	0.058			1.03 (0.93–1.16)	0.559	1.07 (0.95–1.21)	0.279
Pathologic stage	2.05 (1.14–3.69)	0.016					2.03 (1.10–3.77)	0.024
Presence of remission	4.14 (1.52–11.30)	0.006					3.21 (1.02–10.13)	0.047
Treatment	1.80 (0.62–5.25)	0.281					1.54 (0.51–4.64)	0.446

Abbreviations: eGFR, estimated glomerular filtration rate; UPCR, urinary protein to creatinine ratio; cTNFR1, circulating tumor necrosis factor receptor 1; cTNFR2, circulating tumor necrosis factor receptor 2; HR, hazard ratio; CI, confidence interval.

a Clinical parameters (Age, sex, hypertension, eGFR, UPCR, pathologic stage, presence of remission, treatment) were examined with cTNFR1.

b The effects of cTNFR1 and cTNFR2 were examined separately.

We compared the diagnostic performance of cTNFRs, sCr, and urinary protein to creatinine ratio (UPCR) for the determination of renal progression through Receiver operating characteristic (ROC) analysis (Figure 3C). Circulating TNFRs had higher areas under the ROC curves (AUCs) than sCr or UPCR. The AUCs for cTNFR1, cTNFR2, sCr, and UPCR were 0.719 (95% confidence interval [95% CI]: 0.597–0.841), 0.724 (95% CI: 0.599–0.849), and 0.520 (95% CI: 0.375–0.666), 0.607 (95% CI: 0.479–0.735), respectively.

### Variability of anti-PLA_2_R Reactivity/Levels with Circulating TNFRs Levels

**Figure 4:**
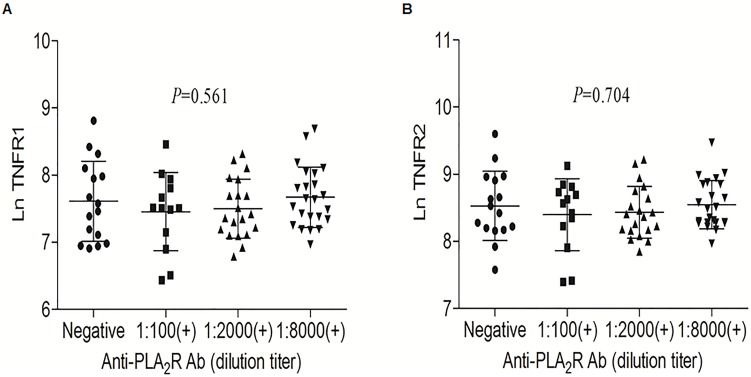
Correlation between the circulating TNFRs levels and autoantibody against phospholipase A_2_ receptor (anti-PLA_2_R). (A) The statistically insignificant relationship between Ln cTNFR1 level and anti-PLA_2_R. (B) The statistically insignificant relationship between Ln cTNFR2 level and anti-PLA_2_R.

A total 72 of 90 patients with nephrotic syndrome were performed western blotting to identify anti-PLA_2_R reactivity/levels. The prevalence of anti-PLA_2_R at the time of initial diagnosis was 77.8% (56 of 72). We analyzed the correlation between the cTNFRs levels and anti-PLA_2_R reactivity/titers. The cTNFRs levels were not statistically different between the patients with anti-PLA_2_R reactivity and those without anti-PLA_2_R reactivity (2070.5±1085.3 pg/mL vs. 1834.1±1519.9 pg/mL for cTNFR1, *P* = 0.398; 5024.4±2159.2 pg/m vs. 4575.6±3228.2 pg/m for cTNFR2, *P* = 0.500). Furthermore, we explored the variations in the cTNFRs levels according to quantitative anti-PLA_2_R levels but found no significant correlation Ln cTNFRs and anti-PLA_2_R titers (Figure 4).

### Renal Expression of TNFRs

**Figure 5:**
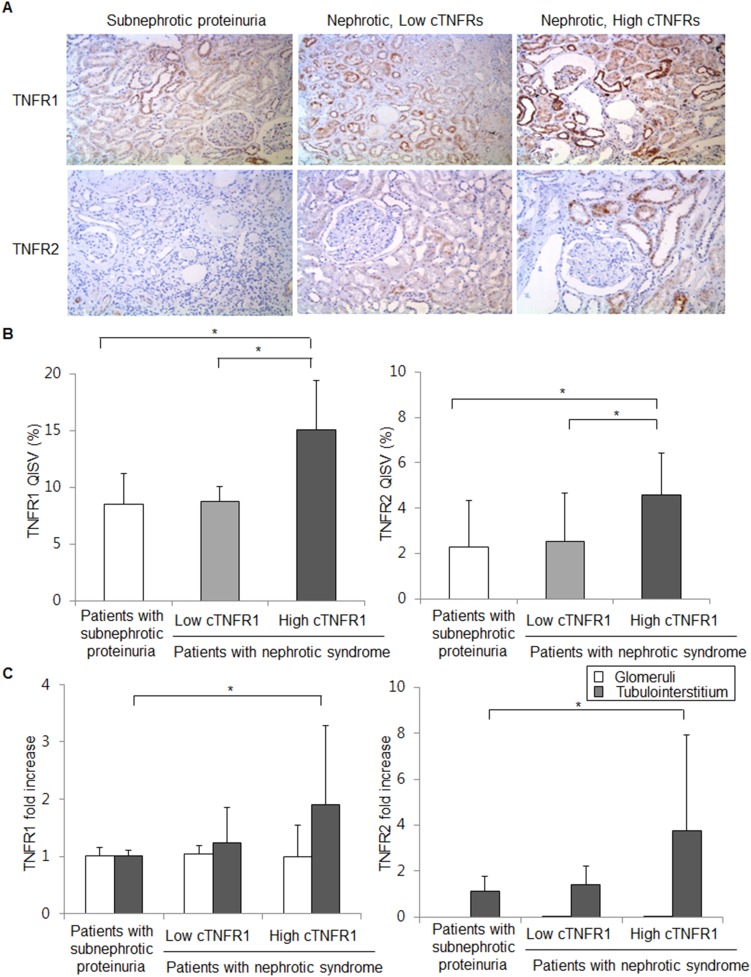
Renal expression of TNFRs according to circulating TNFRs. (A) Immunohistochemical staining of TNFRs expression in paraffin-embedded kidney biopsy sections. TNFR1 is expressed in the glomeruli and tubules in the patients with subnephrotic proteinuria, low cTNFRs levels, and high cTNFRs levels, respectively. TNFR2 is expressed in the tubules but rarely in the glomeruli in the patients with subnephrotic proteinuria, low cTNFRs levels, and high cTNFRs levels, respectively. The intensity of the TNFR2 expression was relatively weaker than that of TNFR1 expression. Original magnification, ×20. (B) Quantitative immunohistochemical analysis of renal TNFRs expression. The quantitative immunohistochemical staining value (QISV, %) was calculated as the integrated optical density divided by the total area occupied by the stained sections in each slide by using computer-assisted quantitative analysis (Qwin3, Leica, Rijswijk, Netherlands). (C) Renal expression of TNFRs in the glomeruli or tubules by performing real-time quantitative reverse transcription-polymerase chain reaction. The Kruskal-Wallis test with Dunn’s method was used. *P<0.05.

Next, we analyzed the renal expression of TNFRs according to cTNFRs levels to evaluate the relationship between the membrane-bound form of TNFRs in the kidney and circulating form of TNFRs. First, renal TNFRs expression was identified by performing immuohistochemical analysis in 28 kidney biopsy specimens from the patients with iMN (9 with subnephrotic proteinuria, 10 with nephrotic syndrome and low cTNFRs levels, and 9 with nephrotic syndrome and high cTNFRs levels). In kidney tissue, TNFR1 was predominantly stained in the glomeruli and tubules (Figure 5A). The quantitative immunohistochemical staining value (QISV) in kidney tissue, including glomeruli and tubules, was significantly higher in the patients with high cTNFR1 level than in those with low cTNFR1 level or subnephrotic proteinuria (*P* = 0.045, *P* = 0.015, respectively; Figure 5B). No significant difference was found between the patients with low cTNFR1 level and subnephrotic proteinuria (*P* = 0.879).

The intensity of TNFR2 expression in kidney tissue was relatively weaker, though detectable, than that of TNFR1 expression. TNFR2 was predominantly expressed in the tubules but rare in the glomeruli. In the patients with subnephrotic proteinuria, TNFR2 was rarely expressed (Figure 5A and 5B). The QISV of TNFR2 was highest in the patients with high cTNFR2 level compared with those with low cTNFR2 level or subnephrotic proteinuria (*P* = 0.043, *P* = 0.023, respectively). Again, no significant difference was observed between the patients with low cTNFR2 level and subnephrotic proteinuria (*P* = 0.817).

In addition, we quantified the renal expression of TNFRs by performing real-time PCR in the glomeruli and tubules of 28 kidney biopsy specimens from the patients with iMN (4 with subnephrotic proteinuria, 6 with nephrotic syndrome and low cTNFRs levels, and 18 with nephrotic syndrome and high cTNFRs levels; Figure 5C). TNFRs expression, especially of TNFR2, in the glomerular compartment was weaker than that in the tubular compartment. Whereas TNFR1 expression in the glomerular compartment was generally similar among the three groups, the TNFR1 expression in the tubular compartment was dominant in the patients with high cTNFR1 level compared with those with subnephrotic proteinuria (*P* = 0.005). Again, significant association in tubular TNFR2 expression was identified between the patients with high cTNFR2 level and subnephrotic proteinuria (*P* = 0.011).

## Discussion

In this study, we found that the cTNFRs levels in the patients with iMN were significantly higher in those with nephrotic syndrome than in those with subnephrotic proteinuria or in the healthy volunteers. Furthermore, the cTNFRs levels at the time of initial diagnosis were associated with renal progression, suggesting that cTNFRs levels at the time of initial diagnosis may forecast the renal progression of iMN. This result seems to corroborate that of recent studies from the Joslin Diabetes Center that increased levels of cTNFRs are strong predictors of renal function loss, including progression to stage 3 chronic kidney disease (CKD) or ESRD in type 1 and 2 diabetic nephropathy [17], [18]. Moreover, the patients with high cTNFRs levels exhibited higher renal expression levels of TNFRs in immunohistochemical staining and real-time PCR. This may indicate that the injured kidney itself could be the source of increased TNFRs expression in kidney disease.

TNFα has an important role in the renal injury cascade [14], [19], [20], [21]. TNFα is a functional 26-kDa homotrimer transmembrane protein and is a dualistic cytokine with proinflammatory and immunoregulatory functions [22]. These functions are relayed by TNFRs, which exist in soluble or membrane-bound forms [23], [24]. Whereas, TNFR1, but not TNFR2, is expressed in the glomeruli in healthy subjects [25], TNFR1 and TNFR2 are expressed in glomerular and tubular cells after renal injury [20]. A large body of evidence explicates the association of TNFRs with inflammatory kidney disease [17], [18], [26], [27], [28], but there is a paucity of data on the correlation between clinical manifestations and cTNFRs levels in patients with iMN.

The mechanism of iMN is non-inflammatory. However, high grade proteinuria due to glomerular disease can stimulate tubular cells to synthesize chemokines such as monocyte chemoattractant protein-1, RANTES and fractalkine [29]. These chemokines can assemble monocytes and T cells that attract neutrophils. Especially, recruited macrophage synthesized several secretory products including TNFα that induce ongoing tissue injury.

In our study, elevated cTNFRs levels at the time of diagnosis were good predictors of renal progression. However, the underlying mechanisms of elevated cTNFRs levels associated with increased risk of renal progression remain unclear. Nevertheless, we found a significant association between cTNFRs levels and histopathological findings. Histological severity tended to worsen with every cTNFRs tertile increment. In turn, cTNFRs levels may reflect the severity of histological findings. The TNFα pathway could activate the production of proinflammatory cytokines and chemokines [30]. It may induce direct renal injury. In addition, the TNFα pathway could activate cellular damage and apoptosis, recruit inflammatory cells, and cause tubulointerstitial changes [31], [32], [33], [34], [35]. Lai *et al.* already identified this hypothesis in IgA nephropathy [16]. They suggested that TNFα production in mesangial cells and podocytes is up-regulated by autocrine mechanisms, and TNFRs are also up-regulated. Up-regulated TNF pathway markers finally lead to renal impairment. In present study, the cTNFRs levels within the highest tertile were associated with an increased risk of renal progression, and the association reached statistical significance after adjusting for several clinical and histological factors. We recently found that cTNFRs levels were higher even in patients with early chronic kidney disease (stages 1 and 2) than in healthy volunteers (manuscript in preparation). This finding suggests that increased cTNFRs levels reflect advanced histological changes, even with stable renal function. Based on the presented data, we assumed that the cTNFRs levels were associated with not only renal injury itself but also renal disease progression.

PLA_2_R has been known to be a major antigen for iMN [9]. We reported that anti-PLA_2_R is strongly expressed in Korean patients and is associated with the clinical disease activity of iMN [36]. Unfortunately, the present study did not show a significant association between the variations of cTNFRs levels and anti-PLA_2_R reactivity. Anti-PLA_2_R was not present in all the iMN patients, even as a specific marker of iMN. While anti-PLA_2_R is detected in human glomeruli, particularly in podocytes [9], TNFRs are predominantly detected in tubular cells after renal injury. Although the finding does not mean that TNFRs are targeted by the immune system, it may suggest the role of TNFRs that could contribute to the progression of iMN.

The present study had some limitations. First, urinary TNFRs levels and other inflammatory markers were not investigated. However, plasma and urinary TNFRs levels were previously measured in experimental animal models and results showed the correlation between plasma and urinary TNFRs levels [37]. In addition, cTNFRs levels were significantly associated with progressive nephropathy in diabetes patients [17], [18]. Therefore, circulating TNFRs levels may be dictate to predict renal progression. Second, the follow-up period was relatively short. Third, although ethnicity can influence several clinical outcomes and PLA_2_R prevalence, the data were only acquired for the Korean population [38], [39]. Fourth, because follow-up samples were unavailable, we did not show how to change the cTNFRs levels after follow up.

Consequently, cTNFRs levels at the time of initial diagnosis might provide useful prognostic information as early biomarkers in patients with iMN with a high risk for renal progression. Further studies are needed to establish the role of cTNFRs as predictors of a specific treatment response.

## Materials and Methods

### Study Subjects and Serum Samples

We performed a prospective study of patients with iMN. For this study, 113 patients with renal biopsy-proven iMN and 43 healthy volunteers were enrolled between January 2002 and June 2012. Patients younger than 15 years or those with secondary MN were excluded. Healthy volunteers were participants in the study of ‘Korean coefficients for glomerular filtration rate estimation by MDRD study equations’ funded by the Korean Society of Nephrology [40], and its validation study funded by the Korea National Enterprise for Clinical Trials. They were defined as those who had no urinary abnormalities and had a systemic inulin clearance greater than ≥60 mL/min/1.73 m^2^. Nephrotic range proteinuria was defined as a UPCR greater than 3.5 g/g Cr.

This study was reviewed and approved by the institutional review boards of Seoul National University Hospital and Yonsei University Severance Hospital in Seoul, Korea. We conducted the study according to the principles of the Declaration of Helsinki and with written informed consent from all the participants.

### Clinical Assessment

Clinical parameters were collected for demographic and laboratory findings at the time of initial diagnosis. The primary outcome was remission defined as either a CR or a PR. CR was defined as a UPCR less than 0.2 g/g Cr [41]. PR was defined as a reduction in UPCR by less than 3.5 g/g Cr or more than 50% from the baseline with stable renal function [42]. Patients with neither of these remissions were defined as non-responders. Renal progression was defined as a decrease in baseline eGFR by more than 30%. Hypertension was defined as a blood pressure of greater than 140 mmHg systolic, greater than 90 mmHg diastolic, or the use of antihypertensive drugs.

### Histopathological Analysis

The pathological findings were interpreted by an experienced pathologist. Each case was classified from stage I to IV according to the Ehrenreich and Churg system [43]. On light microscopy, glomerular sclerosis was scored as the proportion of glomerular sclerosis as follows: 0, no sclerosis; 1, slight or <25% sclerosis; 2, mild or 25–49% sclerosis; or 3, moderate to severe or >50% sclerosis. The degree of tubular atrophy, interstitial fibrosis, and interstitial inflammation was defined as a score of 0 (normal), 1 (slight), 2 (mild), or 3 (moderate to severe) by using semi-quantitative methods [42].

### Measurement of TNFRs

The levels of cTNFR1 and cTNFR2 were determined by performing enzyme-linked immunosorbent assay (ELISA; R&D Systems, Minneapolis, MN, USA) according to the manufacturer’s instructions. The samples were subjected to a duplicate and blind testing. Absorbance was detected with an ELISA reader at 450 nm.

### Measurement of anti-PLA_2_R

We measured reactivity and titers of the anti-PLA_2_R at the time of initial diagnosis by performing western blotting, as previously described [36]. The anti-PLA_2_R titers were classified into four groups as follows: the negative group, defined as the states without detection of anti-PLA_2_R; 1:100 (+) group, defined as the states with detection of anti-PLA_2_R at a dilution of 1:100; 1:2000 (++) group, defined as the states with detection of anti-PLA_2_R at a dilution of 1:2000; or 1:8000 (+++) group, defined as the states with detection of anti-PLA_2_R at a dilution between 1:2000 and 1:8000.

### Immunohistochemical Analysis

After deparaffinization, antigen retrieval was performed in 4-µm thick sections immersed in a 10 mM sodium citrate solution (pH 6.0), by using a microwave oven (650 W, 20 min). Endogenous peroxidase activity and nonspecific binding was blocked by using 3% hydrogen peroxide. The tissue slides were incubated overnight with primary antibodies at 4°C. Rabbit polyclonal antibody to TNFR1 (1:300 dilution; ab19139, Abcam, Cambridge, MA, USA) and rabbit monoclonal antibody directly against human TNFR2 (1:300 dilution; EPR 1653, Novus Biologicals, Littleton, CO, USA) was used as primary antibodies for TNFR1 and TNFR2, respectively. Renal expression was detected by using a HRP-conjugated anti-rabbit antibody (Dako, Carpinteria, CA, USA). Sections were stained with 3,3′-diaminobenzidine solution for 30 sec and subsequently counterstained with hematoxylin. Negative controls were incubated without primary antibody in the same sections.

For TNFRs, a computer-assisted quantitative analysis (Qwin3, Leica, Rijswijk, Netherlands) was performed using the stained sections. In each specimen, 5 randomly selected non-overlapping high-power fields were acquired by using ×20-objective lens and then analyzed by using the Image-Pro Plus software. The QISV was calculated as the integrated optical density divided by the total area occupied by the stained sections in each slide.

### Real-time Quantitative Reverse Transcription-Polymerase Chain Reaction

Tissue samples were stored in an RNase inhibitor (RNAlater, Ambion, Austin, TX, USA) and manually microdissected into glomeruli and tubulointerstitial fragments under a stereomicroscope with two dissection needle holders. RNA was isolated from glomerular and tubulointerstitial fragments with standard methods by using the commercially available SV Total RNA Isolation system (Promega Corporation, Madison, WI, USA) and RNeasy-Mini kit (QiagenGmBH, Hilden, Germany), respectively. Two micrograms of RNA from the glomerular and tubulointerstitial fragments were reverse transcribed by using oligo-d(T) primers and avian myoblastosis virus reverse transcriptase polymerase (Promega Corporation). Real-time reverse transcription-polymerase chain reaction was performed with Assay-on-Demand Taqman probes and primers for TNFR1, TNFR2, and glyceraldehyde 3-phosphate dehydrogenase (GAPDH; Applied Biosystems, Foster City, CA, USA), with the ABI PRISM 7500 Sequence Detection System. All PCR reactions were performed in duplicate. The TNFR1 and TNFR2 expression levels were normalized with respect to the GAPDH expression. The comparative threshold cycle (C_T_) method (also known as the delta-delta C_T_ method) for comparing relative renal expression was applied [44].

### Statistical Analysis

The data were presented as mean ± SD or frequency (count and percentage). The subjects’ characteristics were analyzed by using Student’s *t* test for continuous variables, Chi-squared test for categorical variables, and one-way analysis of variance by using Scheffe’s multiple comparisons for continuous variables among 3 or more groups. The Kruskal-Wallis test with Dunn’s method was used for multiple comparisons for non-parametric data. ROC analysis was used to explore the diagnostic performance of cTNFRs for the determination of renal progression. To identify the cumulative risk for renal progression according to cTNFRs levels, Kaplan-Meier analyses and log rank tests were performed. Furthermore, a multivariate Cox proportional hazards regression analysis using the backward stepwise process was applied to identify the association between cTNFRs levels and renal progression. All the analyses were performed by using the SPSS version 18.0 software (SPSS Inc., Chicago, IL, USA), and statistical significance was defined as *P*<0.05.

## Supporting Information

Figure S1 **Relationship between cTNFR and eGFR or amount of proteinuria.** A. Negative correlation of log-transformed cTNFRs (Ln cTNFRs) level with eGFR (Pearson’s correlation coefficient (*r*) = −0.571, *P*<0.001 for Ln cTNFR1 and *r* = −0.466, *P*<0.001 for Ln cTNFR2), B. Positive correlation of Ln cTNFRs level with amount of proteinuria (*r* = 0.240, *P* = 0.024 for Ln cTNFR1; *r* = 0.196, *P* = 0.066 for Ln cTNFR2).
(TIF)

Data S1 **Raw data of the manuscript.**
(XLSX)
