# Supplementary figures and images for: Circulating TNF Receptors Are Significant Prognostic Biomarkers for Idiopathic Membranous Nephropathy
Source: PLoS One. 2014 Aug 6;9(8):e104354. doi: 10.1371/journal.pone.0104354 (PMC4123977; doi:10.1371/journal.pone.0104354)

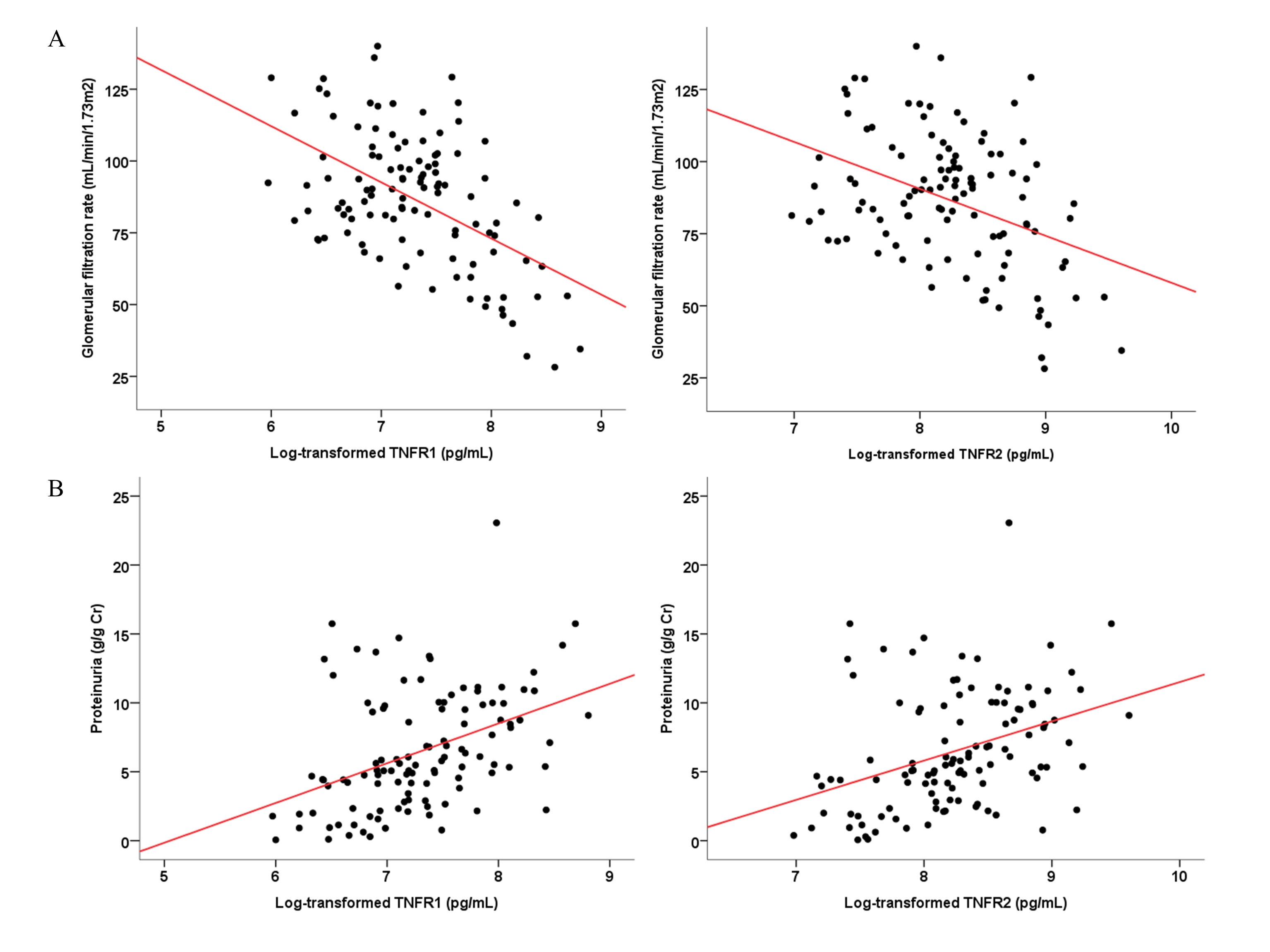

Supplement: Figure S1 — Relationship between cTNFR and eGFR or amount of proteinuria. A. Negative correlation of log-transformed cTNFRs (Ln cTNFRs) level with eGFR (Pearson’s correlation coefficient (r) = −0.571, P<0.001 for Ln cTNFR1 and r = −0.466, P<0.001 for Ln cTNFR2), B. Positive correlation of Ln cTNFRs level with amount of proteinuria (r = 0.240, P = 0.024 for Ln cTNFR1; r = 0.196, P = 0.066 for Ln cTNFR2). (TIF) [file pone.0104354.s001.tif]
